# Global dataset for seized and non-intercepted illegal cheetah trade (*Acinonyx jubatus*) 2010–2019

**DOI:** 10.1016/j.dib.2021.106848

**Published:** 2021-02-08

**Authors:** Patricia Tricorache, Shira Yashphe, Laurie Marker

**Affiliations:** a Illegal Wildlife Trade Expert, Mexico City 11500, Mexico; b Illegal Wildlife Trade Lead, Cheetah Conservation Fund (CCF), Tel Aviv, Israel; c Executive Director, Cheetah Conservation Fund (CCF), Otjiwarongo, Namibia

**Keywords:** Cheetah, CITES, Illegal wildlife trade, Pet trade, Social media, Wildlife trade, Cybercrime

## Abstract

Cheetahs (*Acinonyx jubatus*) are a keystone predator of savanna systems in Africa, yet their populations have dramatically declined due to pressures such as human-wildlife conflict, loss of habitat, and most notably the illegal trade in live cheetah cubs as pets. We provide the most extensive dataset relevant to seized and non-intercepted illegal trade in live cheetahs and cheetah parts for the decade 2010-2019, spanning over 300 sources and 56 countries in Africa, the Middle East, Asia, Europe, Oceania and North America. It includes 1,884 individual incidents involving at least 4,000 cheetahs or cheetah parts or products likely or confirmed to breach national laws or CITES regulations. While the covert nature of illegal trade of any kind makes it extremely difficult to capture its true volume, we believe that the information contained in this dataset demonstrates the need for a more in-depth look into illegal cheetah trade, including sustainability assessments with emphasis in regions where cheetah populations are small and widely exploited, such as the Horn of Africa, as this dataset suggests. Ultimately, such actions could lead to improved enforcement and legal frameworks, and provide a guide for CITES actions involving international cooperation and demand reduction efforts.

## Specifications Table

**Table:** 

Subject	Ecology			
Specific subject area	Ecology and identification of threats			
Type of data	Table			
How data were acquired	This dataset is a compilation of direct communications with field informants, veterinarians, and cheetah owners; open source information including official government and CITES (Convention on International Trade in Endangered Species of Wild Fauna and Flora) reports, databases, reports and media articles; and the authors’ field work. Additionally, we include data obtained through e-commerce and social media platforms and apps where wildlife are offered for sale.			
Data format	Raw and filtered			
Parameters for data collection	This dataset comprises all identified cases of confirmed, alleged and non-intercepted illegal cheetah trade, i.e., seizures and possession/trade, dated between January 1, 2010 to December 31, 2019.			
Description of data collection	Official sources: 1) CITES web site (biennial reports, meeting documents and trade database), which contains data provided by CITES national authorities or Intergovernmental Organizations (IGO), e.g., the United Nations Office for Drugs and Crime. 2) Court records obtained through searching the internet for name(s) of suspects mentioned in media articles, or through periodical searches for the word “cheetah” in web sites that publish court records. Databases: a) International Cheetah Studbooks (ICSB), which contain firsthand reports by cheetah-holding facilities around the world including information on the date a cheetah was received and birth type (captive, wild or unknown). ICSB entries were compared with CITES trade database entries to determine whether a transfer was permitted or reported as a confiscation or seizure (source “I”); and b) The TRAFFIC International Wildlife Trade Portal, which includes open-source instances of illegal wildlife trade. Primary sources: 1) Authors’ field work resulting from assisting governments with the rescue or disposal of seized animals. 2) Direct reports from firsthand witnesses. Secondary sources: 1) Communications with NGO/government staff doing field work in known cheetah trafficking areas and field informants. 2) Open sources, i.e., media articles, blogs and reports. Sellers: Trade advertisements found on eCommerce web sites, social media platforms and mobile phone apps. Sellers can be primary or secondary sources (direct vs partners or advertisers).			
Data source location	Due to the sensitive nature of much of this dataset, it is necessary to protect the confidentiality of key individuals, organizations, and geographical locations, some of which might be an active part of law enforcement efforts. The top 10 countries involved in 96.4% of incidents in this dataset were:			

	Country	Cheetah range	Incidents	Generic Coordinates

	Saudi Arabia	No	51.0%	23.8859° N, 45.0792° E
	UAE	No	12.0%	29.3117° N, 47.4818° E
	Kuwait	No	11.9%	23.4241° N, 53.8478° E
	Somaliland	Yes	7.7%	9.4117° N, 46.8253° E
	Qatar	No	3.5%	25.3548° N, 51.1839° E
	Ethiopia	Yes	2.3%	0.0236° S, 37.9062° E
	Yemen	No	2.3%	15.5527° N, 48.5164° E
	Kenya	Yes	2.2%	9.1450° N, 40.4897° E
	South Africa	Yes	1.9%	30.5595° S, 22.9375° E
	Somalia	Unknown	1.6%	5.1521° N, 46.1996° E
	In addition, data were obtained from multiple open-source web sites including: - CITES (http://www.cites.org) - EAGLE Network (https://www.eagle-enforcement.org/) - Int'l Cheetah Studbooks 2010, 2013, 2016, 2017, 2018 (https://cheetah.org/learn/resource-library/#acc-panel-international-studbooks) - TRAFFIC International Wildlife Trade Portal (https://www.wildlifetradeportal.org/) - TRAFFIC Kenya Report (https://www.traffic.org/site/assets/files/2410/kenya-wildlife-trafficking-report.pdf) - Wildlex (https://www.wildlex.org/search?q=cheetah)			
Data accessibility	Data is provided with this article. Direct URL: https://data.mendeley.com/datasets/84k92j4n3y/2			

## Value of the Data

• This dataset covers 10 years of illegal cheetah trade incidents worldwide, providing the first extensive look into seizures and non-intercepted cases affecting a single species. Over the last century, the geographic distributional range of cheetahs has seen a reduction of over 90%. Today, the known global cheetah population in the wild is estimated at only 7,100 adults and adolescents [1]. Fifteen of 17 range states with national cheetah conservation action plans identify illegal trade as posing a threat to cheetah populations and/or identify activities needed to address illegal trade^1^. The dataset may be of significant value to assess the sustainability of legal and illegal trade in cheetahs –live, products or derivatives, particularly affecting areas of East Africa with low cheetah-population densities [1], e.g., of the subspecies *A. j. soemmeringii*.
• The reports on illegal activities involving cheetahs contained in this dataset will be useful to understanding the global nature, extent, potential trafficking routes, and impact of this trade. This, in turn, could be used by law enforcement agencies, environmental investigative organizations, and non-government organizations working on the ground to promote action and raise awareness.
• This dataset may contribute to further research that can lead to improved enforcement and legal frameworks through international cooperation and demand reduction efforts.

## 1 Data Description

The global dataset for illegal cheetah trade (*Acinonyx jubatus*) presented with this paper, covers the last ten years (January 1, 2010 and December 31, 2019) of seizures, as well as non-intercepted, alleged (asserted without proof) or suspected (believed to exist) illegal cheetah trade incidents known to us worldwide, including live cheetahs or cheetah parts (e.g., skins, skulls) and derivatives (e.g., coats, medicinal products).

Illegality is defined by national laws and international conventions. The United States Endangered Species Act of 1973 effectively banned all trade in wild cheetahs and their products. In 1975, the Convention on International Trade in Endangered Species of Wild Fauna and Flora (CITES) listed the cheetah under its Appendix I category: the most endangered plants and animals. Under Appendix I, wild cheetahs cannot be traded commercially, but captive-bred cheetahs can be traded by facilities registered with CITES [2]. Only two such facilities exist, both in South Africa. In addition, CITES designates special export quotas for live cheetahs and hunting trophies, which are subject to Appendix III provisions. Currently, quotas exist for Namibia (150), Zimbabwe (50) and Botswana (5). Neither Appendix II nor III require import permits [3].

All countries included in this dataset are Parties to CITES, except for Somaliland. Somaliland is a self-proclaimed autonomous region of Somalia with its own government institutions. As it is a legal jurisdiction of critical importance to combatting illegal trade of cheetahs, it is included as a separate entity under countries for the purposes of this dataset.

This dataset was compiled utilizing over 300 sources, including direct communications with field informants, veterinarians, and cheetah owners; open-source information including official government and CITES (Convention on International Trade in Endangered Species of Wild Fauna and Flora) reports, databases, reports and media articles; and the authors’ field work. Additionally, we include data obtained through e-commerce and social media platforms and apps where wildlife is offered for sale.

The dataset includes 1,884 individual incidents involving at least 4,184 cheetahs (87.1% live, 12.9% parts or derivatives). The data spans 56 countries, 15 of which are cheetah-range countries assumed to play the role of source or transit countries, with Somaliland (42.4%), Kenya (12.7%), and Ethiopia (10.2%) being the most represented in terms of cheetah units. Iran, with a remaining population of less than 50 cheetahs [4], recorded three cases involving three cheetahs during the decade of this research. The remaining 41 countries are non-cheetah range considered transit or destination countries, with Saudi Arabia (60.5%), Kuwait (14.2%) and UAE (13.7%) being the most represented. The dataset includes 2,316 online advertisements posted during this study's timespan, representing 528 sellers and 1,430 different offers involving 2,298 cheetahs suspected as wild sourced. Advertisements were found on social media (88.4%); e-Commerce (7.9%); and phone apps (3.7%). The top 10 platforms where advertisements for cheetahs were found are listed on Table 1.

**Table 1:** Top 10 platforms identified as being used for advertising cheetahs.

Platform type	Platform name	Advertisements recorded	% of total
Social media	instagram.com	1736	75.0%
eCommerce	haraj.com.sa	142	6.1%
Phone app	4sale	73	3.2%
Social media	youtube.com	65	2.8%
Social media	twitter.com	63	2.7%
Social media	facebook.com	45	1.9%
Social media	ma7room.com	21	0.9%
eCommerce	mzadqatar.com	14	0.6%
eCommerce	harajanimals.com	13	0.6%
Social media	ourpetclub.com	11	0.5%
	Total	2183	94.2%

While all advertisements were harvested from open sources/public accounts, special care was taken to ensure that no data use policies were breached. Consequently, any identifiers that could reveal, or lead to revealing, information about subjects linked to this research subjects were anonymized. This procedure was also followed to protect the identity of individual sources or people linked to listed incidents (see Ethics Statement).

The covert nature of illicit activities makes it difficult to identify every instance. Hence, this dataset is likely to capture only a fraction of the actual trade while the numbers of traded cheetahs may be significantly larger.

## 2 Experimental Design, Materials and Methods

Decades of work researching threats facing cheetahs had suggested that one of these threats was the illegal trade in cheetahs, mainly for their parts and derivatives. In 2005 we received our first direct report of live cheetah cubs being sold illegally in a remote area of Ethiopia and coordinated their confiscation. Stemming from this incident, as well as the decrease in wild cheetah populations across their range [1], we saw the need to estimate the volume of trade in cheetahs. We began to systematically record all instances of illegal trade accessible to us. As our networks expanded over time, data obtention became more consistent, either through direct reports or our involvement to support enforcement authorities with handling, placement and disposal of seized specimens.

Thus, this dataset consists of information obtained through our field work or through networks set up on the field. We also compiled data reported by government representatives and CITES, databases, field informants, veterinarians, cheetah owners, rescue facilities, and media articles. Additionally, we included data obtained through e-commerce and social media platforms where wildlife are offered for sale [5], [6], [7]. In all cases, every effort was made to prevent duplications of incidents gathered from different sources. Consequently, we compared all available information such as dates, locations, number of cheetahs, people involved and description of each incident to identify any potential similarities. Where duplications were suspected, incidents were merged into one.

Given the covert nature of illegal activities, and that this is the first research of its kind in terms of timespan and multiplicity of sources, we approached this research in an organic manner, i.e., we searched all sources of information available to us through the methods described below. In most cases, each search led us to additional sources, which were included going forward.

Our sources were classified as follows and each was processed based on the methodology described in sub-section 1. Processing the sources.

**Table:** 

Source type	Description
Official	CITES’ publications: trade database, commissioned studies, meeting documents; court records; communications with government entities.
Databases	International Cheetah Studbook (ICSB), TRAFFIC International Wildlife Trade Portal.
Primary sources	First-hand observations; wildlife rescue facilities; cheetah owners and veterinarians; field researchers/surveys; and witnesses.
Secondary sources	Open or second-hand sources, e.g., media articles, non-governmental organizations (NGOs) or NGO/government partnerships’ reports, blogs; field informants.
Sellers	Trade advertisements found on eCommerce web sites, social media platforms and mobile phone apps. Sellers were included as a separate category as they can be primary or secondary sources (direct vs partners or advertisers).

To protect the privacy of informants or presumed “Persons of Interest”, all details that might reveal individuals’ personal information were omitted from this dataset and were assigned codes.

### 2.1 Processing the sources

#### 2.1.1 Official sources

All records from official sources were assumed to be true. These include CITES web site documents (e.g., Parties’ biennial reports, meeting documents and trade database), all of which contain data from CITES national authorities or Intergovernmental Organizations (IGO) such as the United Nations Office for Drugs and Crime.

Court records were obtained either through internet searches when the name or names of arrestees were mentioned in media articles, or through periodic searches for the word “cheetah” in web sites that publish court records.

Direct reports from government or enforcement officials which lacked evidence of a seizure, such as specific details of disposal or official documentation, were further researched online or through personal interviews to avoid including erroneous information. Additional verifications were also performed if confusion on the species in question was suspected, e.g., leopards, servals or caracal cubs reported as cheetahs, or leopard skins reported as cheetah skins, by searching for original images.

#### 2.1.2 Databases

In addition to the CITES trade database (see 1.1 Official Sources), we consulted two reliable databases: International Cheetah Studbook (ICSB) and TRAFFIC International Wildlife Trade Portal, which are maintained by reputable organizations.

The ICSB contains firsthand reports by cheetah-holding facilities around the world. Facilities provide information about the date when they received a cheetah and birth type (captive, wild or unknown). Factors such as whether the facility is in a cheetah-range country or not, whether the birth type is unknown or of wild origin, and the circumstances of a transfer (capture, unknown) were considered. We focused on transfers of wild-born or unknown birth type cheetahs into facilities in non-range countries and compared them with CITES trade database entries to determine whether a transfer was permitted or reported as a confiscation or seizure (source “I”). ICSB entries were also cross-checked with seizures reported by CITES national authorities through other means such as CITES questionnaires or meeting reports.

The TRAFFIC International Wildlife Trade Portal is a database that includes open-source instances of illegal wildlife trade. Data from this database were also compared to other sources, since some incidents sourced from this tool included records from the CITES database or Management Authorities’ biennial reports, as well as media articles. We attempted to confirm the latter by cross-checking with other sources, such as court records or police reports, and graded each incident accordingly (see 1.6. Incident Grading).

#### 2.1.3 Primary sources

Our field work data are based on firsthand incidents consisting mainly of helping governments with the rescue or disposal of seized animals. Data based on this work are rated true. Through field work we are often approached by cheetah dealers. Working alongside authorities, we attempt to obtain physical evidence of the existence of cheetahs (images, videos or in-person visits). We maintain detailed records and evidence of all such incidents, including those where seized cheetahs are placed under our care.

Similarly, we are commonly approached by rescue facilities, veterinarians or owners seeking advice for cheetah care. In such cases we conduct informal interviews to obtain information on the animals, as well as images or videos to better assist them and as evidence.

Field informants are an important source of data. Over the years we built a network of confidential informants who either have ties with exotic animal dealers or live in areas where cheetahs are commonly trafficked or sold. We attempt to obtain evidence of all reports from confidential informants who have personally seen or come across a case of actual or potential illegal cheetah trade. In addition to their credibility, informants’ reports are graded based on the level of detail provided, as well as evidence such as images or videos. Finally, field research, firsthand media and investigative reports are also included in this category.

#### 2.1.4 Secondary sources

In this category we include research papers, second-hand media articles, and direct reports from people or organizations not directly involved in an incident, e.g., NGO/government staff doing field work in known wildlife trafficking areas, who often come across reports of illegal cheetah trade. Every effort was made to confirm reports by secondary sources with follow-up searches for official records, substantiation with images or video, and evidence of their primary source including declarations by government officials in the case of seizures reported by media.

We included in our dataset the result of thousands of internet searches over the 10-year period. We set up Google Alerts with the search terms “illegal wildlife”, “pet trade”, “cheetah” AND “illegal”, “pet cheetah”, “cheetahs” AND “trade”, “cheetah” AND “confiscated” and “cheetah trafficking.” An average of 10 alerts were received by email daily and included mainly media articles, blogs and Facebook posts.

We reviewed relevant items to assess their veracity utilizing criteria such as timeliness, level of detail, original versus stock images and declarations by government officials that included name and title. Further internet searches for the same topic were made to find the primary source of an article using keywords from the initial article or specific names of arrestees when available. The word “cheetah” in foreign languages was used to locate additional information relevant to specific countries, e.g., *jagluiperd* (Afrikaans),  (Amharic), شيتا (Arabic), *guépard* (French), *loài báo* (Vietnamese), یوزپلنگ (Persian), *harimad, harimacad* or *harima'ad* (Somali), *guepardo* (Spanish/Portuguese). Where arrests were reported, online court records were searched periodically. Original images were particularly important as in some cases leopards or other cat species were mistaken for cheetahs, whether due to translation or confusion between species.

#### 2.1.5 Sellers

Exotic pets are very popular in the Middle East [8,9]. By 2014, hundreds of pictures on social media depicted people with their pet cheetah or other exotic animals in the Arabian Peninsula [10]. In 2015, following a widely publicized report, “The Illegal Big Cats of Instagram” [11], we began performing extensive searches in multiple languages on Google, social media and eCommerce platforms to identify cheetah advertisements. Since 99% of search results were found in Arabic, we mainly focused on Arabic terms commonly used by sellers: للبيع (for sale), شبل (cub), السوم or سعر (price), بكم (how much), شيتا (cheetah), فهد (leopard – alternate term for cheetah), اتساب ال or اتس اب (WhatsApp), and hashtags using combinations of the same words, e.g., شيتا_للبيع# (cheetah_sale), شبل_للبيع# (cub_sale). We also scanned for numerals commonly used for prices or phone numbers: ١٢٣٤٥٦٧٨٩٠ (1234567890).

Additionally, we monitored sellers’ accounts that were brought to our attention by confidential informants who had dealt with them or are part of their networks. One of these informants provided us with Instagram usernames of the five largest sellers in the UAE. Posts on these accounts yielded hundreds of new discoveries given that sellers often post comments on other sellers’ posts to publicize that they, too, have cheetahs for sale. These were also added to the research.

Our research into online advertisements goes beyond 2010 and has yielded over 60,000 files (JPEGs, PDFs) that include other CITES-listed species. To protect their privacy, advertisers and URLs to their posts were anonymized and assigned unique codes to avoid identifiers that could lead to revealing personal information.

We set up a dedicated account on Instagram, which was found to be the most used platform to advertise cheetahs during this research, to follow posts; however, we did not interact with the users.

The second most used platform found through this research is the Saudi eCommerce web site haraj.com.sa, followed by the Kuwaiti phone app 4sale, both popular for buying and selling multiple items including animals. A Kuwaiti collaborator monitored 4sale through random searches for 35 months (December 2012 to October 2015) and provided us with screen captures of all cheetah offerings found. Although there are other mobile phone apps used in the Gulf countries, these were not monitored during the dataset period as no local SIM card was available.

During our searches we identified accounts that shared the same telephone/WhatsApp numbers. These accounts were merged and cataloged under the listed numbers. The numbers were further searched on Google to identify real names, location, email address, as well as additional advertisements and information on other businesses or accounts connected to them. Every detail found through these searches was recorded. All identifying data was anonymized after collection.

Special care was paid to avoid recording scam posts, i.e., animals do not exist, but money is required as a deposit. To this end, we used reverse Google Image Search to ensure that images were original, as most scammers utilize pictures previously published by others. We searched Google for telephone numbers and email addresses and looked for indicators that are common with legitimate sellers, like wording and pictures or videos of them with the animals.

We found that cheetahs are occasionally advertised in more than one account with the same or similar images but different telephone/WhatsApp numbers. We strived to avoid duplications by performing cursory manual comparisons of over 60,000 images from different accounts saved in our archives by sorting them by similar dates, prices, description of the cheetahs or common denominators, such as furniture and carpeting or jewelry, and conditions in which the animals were showcased. We merged similar advertisements and recorded the earliest publication date with all telephone/WhatsApp numbers and URLs in connection with the same advertisement.

Short advertisements were screen captured, while longer ones were saved as PDF files. All were assigned a filename consisting of the date they were posted (YYYY-MM-DD) and a brief description of the post, e.g., 3 2-mo cubs [price], with their URLs added to the file properties. In the case of advertisements containing videos, we saved those that were more descriptive of the advertisement than the displayed image, e.g., number of cheetahs offered were only evident on the video. The saved files were then recorded in a spreadsheet with all relevant information: URL(s), user ID and telephone/WhatsApp number(s), and a Google translation of the post. While Google translations are poor, they provide enough information to determine the nature of a post.

For the most prolific sellers who post frequent advertisements, we made every effort to compare markings on the cheetahs to minimize the risk of double-counting them. Due to the frequency with which some sellers delete posts, make their pages private or close them to reopen new ones, we downloaded entire Instagram pages of some of those sellers who appeared more active to facilitate searches and preserve evidence.

We have noticed a decrease in the number of online advertisements in some of the Gulf States in the last three years, possibly due to growing regulations on the possession of predators in the Gulf States, e.g., a 2017 UAE law prohibiting private ownership of exotic and dangerous pets without a license. Sellers still using open-source platforms avoid including the word “sale” or prices. Instead they respond to sales enquiries by inviting contact through WhatsApp or private message. Consequently, advertisements that include a telephone/WhatsApp number or ask to be contacted privately were considered offers to sell and were included in this dataset.

Many sellers began avoiding the internet altogether or use apps such as SnapChat, where posted videos disappear after being viewed. Furthermore, sellers have formed chat groups on WhatsApp –an end-to-end encrypted app; membership is approved only for known buyers or sellers and direct referrals. Reports from a confidential source belonging to one of these chat groups were included in the dataset. Based on information provided by this confidential source, sellers are highly suspicious of being contacted by someone with whom they have never dealt before, and more so, by someone who does not speak Arabic. As such, we made the decision of not attempting to infiltrate their networks directly and instead focus on gathering data.

### 2.2 Recording the information

Given our specific aim to quantify the volume of illegal cheetah trade, we adapted the fields in our dataset from various methods utilized by TRAFFIC, the Wildlife Conservation Society, the International Fund for Animal Welfare, and the Wildlife Justice Commission, with whom we have collaborated and shared data over the years. Each incident of actual, alleged or suspected illegal cheetah trade was entered into an Excel spreadsheet organized into six sections described below.

#### 2.2.1 Incident identification, type of incident and dates

Each incident in this dataset was assigned an ID number composed of the researcher's initials, the year when the incident occurred, and a 3-digit number for each incident, e.g., PT-2018-XXX. For this research we selected two types of incidents:

1. Seizures: Cases where cheetahs were reported as taken/seized by authorities
2. Possession/trade: Alleged or suspected cases of cheetahs being held illegally and not intercepted, including those offered for sale or pets reported directly by the owners. However, cheetahs displayed as pets on social media are not included as these could be a duplication of cheetahs advertised for sale.

**Table:** 

Incident ID	Incident type	Incident date	Discovery date	Day (+/-)	Month (+/-)
Assigned by researcher	Possession/trade Seizure	DD-MMM-YY	DD-MMM-YY (date when incident was discovered by researcher)	Applies when exact date of incident is not known	

#### 2.2.2 Location information

All incidents were recorded by the country where they occurred. Countries were assigned role codes (i.e., origin O, transit T, destination D) based on their location and the incident description. Roles could be a combination of two when one could not be assigned with certainty, e.g., origin and/or transit (O/T) or transit and/or destination (T/D). In the decision process we considered whether countries were in cheetah range as well as empirical deductions. When countries of origin, transit or destination were known, these were entered in the corresponding fields. In all cases where a report included a description of the location, this was entered under location type.

**Table:** 

Country role	Geographic region	Country	City/ region	Location type	Origin	Transit	Destination
O = Origin T = Transit D = Destination				E.g., airport, residence	Entered when known		

#### 2.2.3 Incident grading

Each incident was graded based on TRAFFIC guidelines, which were adapted to the specific characteristics of incidents reported in this dataset. Grading is assigned as follows:

**Table:** 

Report grading	Description
A = True	Used when there is no doubt as to the authenticity and reliability of the source or verification means used to corroborate it, e.g., digital evidence, official records, as well as incidents witnessed by the research team.
B = Known and/or firsthand source	Related to a source which has, in most instances, proved reliable, and/or provided firsthand information. This could include police officers, regular informants who have proven reliable previously, rescue facilities and witnesses.
C = Corroborated by secondary means	Used when the source is unknown or did not provide firsthand information, but the incident has been confirmed by secondary means.
D = Deemed credible	Used when the information provided reflects details deemed accurate. It may also be used for incidents involving known individuals, e.g., a known seller.
E = Unable to Judge	Used when it is not possible to make an informed decision based on the available evidence, e.g., confusion with species involved (cheetah vs leopard) or the inability to corroborate the information through other sources.
F = Suspected false	Generally, not included in this dataset, but refers to information which is known or believed to be false. It may be possible, however, to make use of the information if it helps us to understand criminal relationships.

These gradings were based on the ultimate means of verification, which included searching for original photos or video and official records, whether a seller is well known, or the reliability of a source, among others.

**Table:** 

Report grading	Means of verification
A = True B = Known and/or firsthand source C = Corroborated by secondary means D = Deemed credible E = Unable to Judge F = Suspected false	E.g., Photo and/or video, official records, source reliability, known sellers, image search.

#### 2.2.4 Sources

Reliability of a source is an important element to define whether an incident requires further clarification, especially in the cases where the source is not directly involved in the incident (i.e., secondary source). We periodically searched the internet to answer questions regarding the validity of recorded incidents, i.e., court records or databases. As such, we included fields to describe the original source of each report, as well as the ultimate source used to verify an incident.

Reliability ratings were assigned only to the ultimate source, which on occasions was the same as the original source, based on the following factors: known or unknown source, direct experience with a source, timeliness of a report and details/evidence provided.

**Table:** 

Ultimate source type	Ultimate verification source	Ultimate source reliability	Orig. source type	Medium	Original source
A = Official B = Database C = Primary D = Secondary E = Seller	E.g., photo, video, official records, site visit	A = always B = mostly C = fairly D = sometimes E = unreliable F = unknown	A = Original research B = Official C = Open source D = Direct Report	e.g. Email, phone, report, database	Name or identifier

#### 2.2.5 Description of incident

This section includes a full description of an incident, including the number and type of specimens involved (in full cheetah units), whether they were seized or surrendered, number of animals known to be alive or death, including those that died from trade-related consequences (e.g., malnutrition, wounds) after confiscation. Cheetah products and parts are classified as dead. If the number of cheetahs was not specified or deducible due to the nature of the product (e.g., teeth, claws, pieces or bushmeat), we entered UNK. All cheetahs for which details on disposal or fate were unknown were listed as LTF, or lost to follow up. Live cheetahs offered for sale are considered LTF unless a seller specifies that the cheetah died or was sold. Sold cheetahs were classified as alive.

**Table:** 

# Cheetahs	Units	Incident description	Confiscated	Surrendered	Alive	Dead	LTF
If known and in full cheetah units.	Bushmeat Claws Head Live Other Skin Skin pieces Skull Stuffed Teeth Trophy	Includes code numbers for advertisements	# of units if known.	# of units if known.	# live units at or following confiscation if known.	# death units at or following confiscation if known.	# units lost to follow up or unknown fate.

#### 2.2.6 Actions and outcomes

The last section of the dataset includes actions taken by the authors or reported by sources, such as circumstances of an arrest. Known outcomes, such as court decisions and disposal of the animals are entered in the relevant field. In seizure cases, we recorded the detecting agency and the Person of Interest (POI) when reported. Each person involved in an illegal cheetah trade incident was anonymized and given a POI code, while their actual details were kept confidential as part of the Limited Dataset (LDS) (see Ethics Statement). We also included a column for other details such as URLs (e.g., media article, open-source documents, court, or government web sites, etc.) where information was found or confirmed.

**Table:** 

Action taken	Outcome	Detecting agency	POI codes	Other info
			Suspects, arrestees	URLs

## Ethics Statement

This dataset includes data collected from open-source social media and eCommerce platforms that has been fully anonymized. It does not include Limited Data Sets (LDS). LDS consist of any information about the people reporting or involved in an incident or online advertisement, and which can reveal names, telephone numbers, account IDs or any other personal information. These have been assigned identifying codes for the purpose of this dataset. Consequently, all data obtained from the internet complies with data redistribution policies from the platforms.

## Declaration of Competing Interest

No funding was received for this research. The authors declare that they have no known competing financial interests or personal relationships which have, or could be perceived to have, influenced the work reported in this article.

## Footnotes

1 *National Conservation Action Plans for Cheetah and African wild dogs produced in Kenya (2007), Botswana (2007; review 2019), Ethiopia (2010), South Sudan (2009), Zambia (2009; review 2018), Zimbabwe (2009; review 2018), South Africa (2009), Benin (2014), Niger (2012); Chad (2015); Tanzania (2013); Malawi (2011); Namibia (2013); Algeria (2015); Angola (2016); and Burkina Faso (2016) identify trade in live cheetah or their parts as posing a threat to wild populations and/or include targeted activities to combat such trade. Only Zimbabwe (2009; review 2018) and Mozambique (2010) make no such mention of trade, neither is trade in cheetah mentioned in the Strategic Action Plan for Large Carnivore Conservation in Uganda (2010).*
